# Insecticide susceptibility status of *Anopheles gambiae* (s.l.) in and surrounding areas of Lake Tana, northwest Ethiopia

**DOI:** 10.1186/s41182-023-00497-w

**Published:** 2023-01-13

**Authors:** Fasil A. Kendie, Melaku Wale, Endalkachew Nibret, Zena Ameha

**Affiliations:** 1 Department of Biology, Bahir Dar University, P.O. Box 79, Bahir Dar, Ethiopia; 2 Biotechnology Research Institute, Bahir Dar University, Bahir Dar, Ethiopia; 3 Amhara Public Health Institute, Bahir Dar, Ethiopia

**Keywords:** *An*. *gambiae* s.l., Insecticides, Malaria, Resistance, Knockdown, Ethiopia

## Abstract

**Background:**

Vector control is the most effective malaria control and prevention measure. Among these, IRS and LLINs are the most important chemical insecticide interventions used in malaria prevention and control strategies in Ethiopia. However, the long-term effectiveness of these strategies is under threat due to the emergency and spread of insecticide resistance in the principal malaria vector*.* Therefore, this study was carried out, under standardized laboratory conditions to assess the killing effect of some insecticides against *An*. *gambiae* s.l.

**Methods:**

Mosquitoes in late instar larvae and pupae stages were collected from different breeding habitats of the study sites using a soup ladle (350 ml capacity). The immature was reared to adults at optimum temperature and humidity in a field insectary using the WHO protocol. Four insecticides representing three chemical classes were used against adult mosquitoes. These were permethrin, deltamethrin, pirimiphos-methyl and bendiocarb. Susceptibility tests were carried out from September to December 2021 using the WHO standard procedures. Mortality rate, variation, interaction effect and knockdown times (KDT50 and KDT95%) were computed using descriptive statistics, multivariate analysis of variance and log-probit regression model using SPSS version 20 software.

**Results:**

Totally, 1300 *Anopheles gambiae* s.l. were tested to determine the susceptibility status to the four insecticides. Among these, 90.7% of them were susceptible to insecticides, whereas the remaining 9.3% of specimens were resistant to the insecticides. The results of the analysis of variance showed that mortality significantly varied between insecticides (*F* = 26.06, *DF* = 3, *P* < .0001), but not between study locations (*F* = 1.56, DF = 3, *P* = 0.212). On the other hand, the mean comparison of dead mosquitoes showed some signs of interaction between bendiocarb and locations, but not other insecticides and locations.

**Conclusions:**

This study revealed that the knockdown times and effectiveness of different insecticides varied in different study sites. Therefore, insecticide resistance information is very essential for concerned bodies to make informed and evidence-based decisions on vector control.

**Supplementary Information:**

The online version contains supplementary material available at 10.1186/s41182-023-00497-w.

## Background

Chemical insecticides are crucial for controlling vectors in the public health sectors [1]. However, due to extensive and repeated use of insecticides as well as characteristics of insect vector species, resistance was developed in medically important insects, such as major malaria vectors [2]. Resistance is defined as the ability to tolerate an insecticide dosage that would kill the majority of individuals in a typical natural population of the same species [3]. The insecticide susceptibility status of malaria vectors varies in season and it affects the effectiveness of both indoor residual spraying (IRS) and Long lasting insecticide-treated nets (LLINs) vector control methods [4].

The emergence of insecticide resistance in malaria vectors has posed a severe threat to malaria control efforts [5]. All five of the pesticide classes that the WHO recommends are susceptible to resistance such as organochlorines, organophosphates, pyrethroids, carbamates [6] and pyrroles [7, 8] that have been used for IRS and ITNs in main African malaria vectors (*An. gambiae* s.l. and *An. funestus*) [9]. Pyrethroids in the form of ITNs and IRS were regarded to play a significant role in malaria control measures when DDT was removed as the insecticide choice in many areas [5]. However, in Ethiopia, IRS is administered using pirimiphos-methyl, propoxur, and bendiocarb, while LLINs contain deltamethrin [10]. Pyrethroid resistance hinders the effectiveness of control measures, such as ITNs and IRS in regions, where it has been detected [11].

In Ethiopia, more than forty species of *Anopheles* mosquitoes have been recorded and documented [12–14]. Of these, *An*. *arabiensis*, a member of *An*. *gambiae* complex is the principal vector of malaria followed by *An*. *pharoensis*, *An*. *funestus* and *An*. *nili* [15, 16]. Vector control is the most effective malaria control and prevention measure. There are several types of strategies being used to control malaria in endemic regions, but chemical pesticides continue to be the most important [17]. Among these, IRS, insecticide-treated nets (ITNs) and LLINs are the most important in malaria prevention and control strategies in Ethiopia [18]. However, the long-term effectiveness of these strategies is under threat due to the emergency and spread of insecticide resistance in the principal malaria vector, *An. arabiensis* [19].

The major malaria vector has evolved resistance to all five chemical classes of insecticides authorized for IRS and LLINs in Ethiopia [20–22]. However, the levels of susceptibility/resistance of the *Anopheles* mosquitoes varied in different seasons, years and agro-ecological zones [23]. The West African knockdown resistance mutation has been reported in *An. arabiensis* populations at high frequencies [5, 24, 25]. Other successive studies have also recognized the occurrence of similar mutations in malaria vectors in different parts of the country [20, 25].

Knowing the susceptibility level of vectors is very important to select environmentally friendly and effective insecticides to control malaria vectors. As a result, continuous monitoring is critical for preventing the development of insecticide resistance [26]. Nevertheless, a detailed investigation of *Anopheles* mosquitoes and their insecticide resistance level is very few in Ethiopia in general and in Amhara Regional State around Lake Tana in particular. Hence, this study was carried out, under standardized laboratory conditions to assess the killing effect of some insecticides against major malaria vectors (*An*. *gambiae* s.l.).

## Materials and methods

### Study area description

This study was conducted in different locations of Lake Tana, and its surrounding areas, in northwest Ethiopia. Lake Tana is the source of the Blue Nile and is the largest lake in Ethiopia, which contributes up to 60% of the Nile’s water, and 50% of the country’s freshwater. The lake is located in Amhara Regional State at latitude of 11° 36ʹ N, and a longitude of 37° 23ʹ E.

**Fig. 1:**
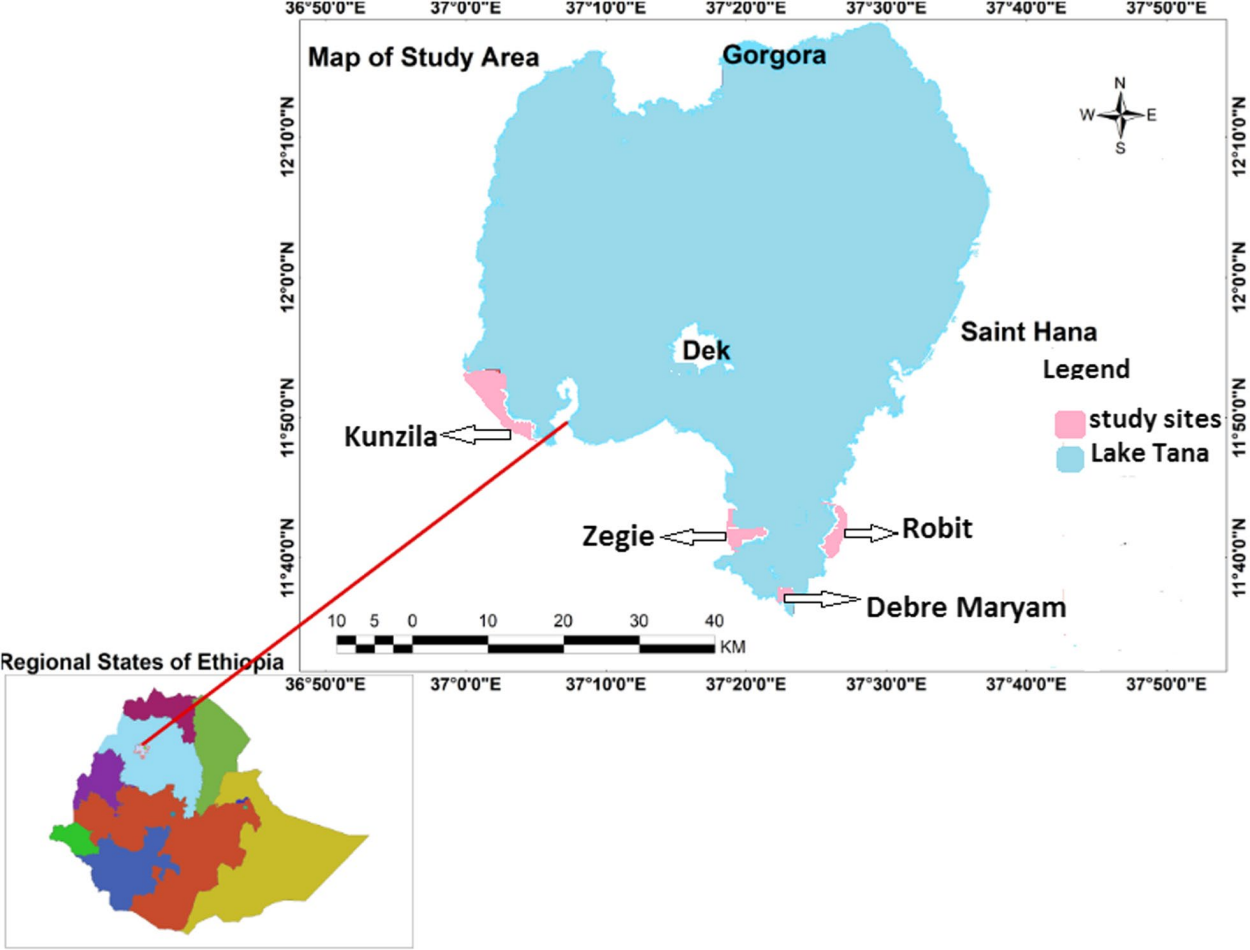
Map of the study area showing four study sites in and around Lake Tana, northwest Ethiopia

For larvae and pupa sampling, the four study sites, which are found in the two districts (Semien Achefer, and Bahir Dar Zuria), and one city administration (Bahir Dar) were selected based on accessibility, suitability, malaria case report, and proximity of areas to the local inhabitants. The data were collected from one island (Debre Maryam), one peninsula (Zegie), and two surrounding mainland areas (Kunzila, and Robit) (Fig. 1). Larvae sampling was done in wetlands associated with Lake Tana.

### Mosquito collection and rearing

Mosquito larvae (late instar) and pupae were collected from different breeding habitats of the study sites using a soup ladle (350 ml capacity). The immature was reared to adults in an insectary prepared in the field using the WHO protocol [27]. They were reared at optimum temperature (25 ± 2 °C) and relative humidity (70–80%), which was maintained using tarpaulin sheets and water-filled buckets, respectively, around the rearing rooms. The larvae were reared in distilled water and fed powdered yeast (Vital Brewer’s Yeast). Then, 2–5 days, non-blood-fed adult females (*An*. *gambiae* s.l.), identified using morphological key [28], were exposed to discriminating dosages of insecticides.

### Insecticides used

Four insecticides representing three chemical classes were used at the following discriminating concentrations against adult mosquitoes: pyrethroids, permethrin (0.75%) and deltamethrin (0.05%), organophosphate, pirimiphos-methyl (0.25%), and carbamate, bendiocarb (0.1%) [29]. These insecticides were chosen based on their current operational importance in the Ethiopian malaria control program [30]. These insecticide-impregnated test papers were obtained from the WHO supply, and distributed through the US President's Malaria Initiative (PMI) vector-link Ethiopia project.

### Insecticide susceptibility tests procedures

Susceptibility tests were carried out in the peak mosquito breeding season between September and December 2021 using WHO adult mosquito bioassay protocols [27]. In the WHO tube tests, 20–25 unfed female *An. gambiae* s.l. were exposed to each insecticide-impregnated paper for an hour at the ideal temperature and relative humidity, which was maintained by placing a moist towel on top of the boxes holding tubes. The numbers of knockeddown mosquitoes were recorded at 10, 15, 20, 30, 40, 50 and 60 min. After exposure periods, mosquitoes were moved into holding tubes and provided with cotton wool soaked with 10% sucrose solution. Death rates were recorded after 24 h of exposure times. The two-control replicate consisted of about 40–50 mosquitos exposed to paper impregnated with olive oil (for bendiocarb and pirimiphos-methyl) and silicone oil (for permethrin and deltamethrin).

If mortality in the control group was less than 5%, no correction of test results is necessary, whereas mortality greater than or equal to 5% requires correction [27]. Abbott's formula was used to correct mortality rates ranging from 5% to 20% [31].

### Data analysis

Mortality in the ranges of 98–100%, 90–97% and less than 90% indicates susceptibility, suggestive existence of resistance and confirmation of resistance genes in the test population, respectively. The observed and corrected percentage mortality were calculated using WHO test guidelines [27]. Resistance and mortality rates were computed using descriptive statistics. The variation and interaction effects of *An*. *gambiae* s.l. death within study sites and insecticides was determined using the multivariate analysis of variance (Two Way MANOVA). The time (in a minute) required to obtain 50% and 95% knockdown in the tested mosquitoes (KDT50 and KDT95%) were determined using the log-probit regression model. Statistical analyses were performed using SPSS version 20 software (SPSS Inc, Chicago, IL, USA) with a level of a significant set at a *p* value less than 0.05.

## Results

**Fig. 2:**
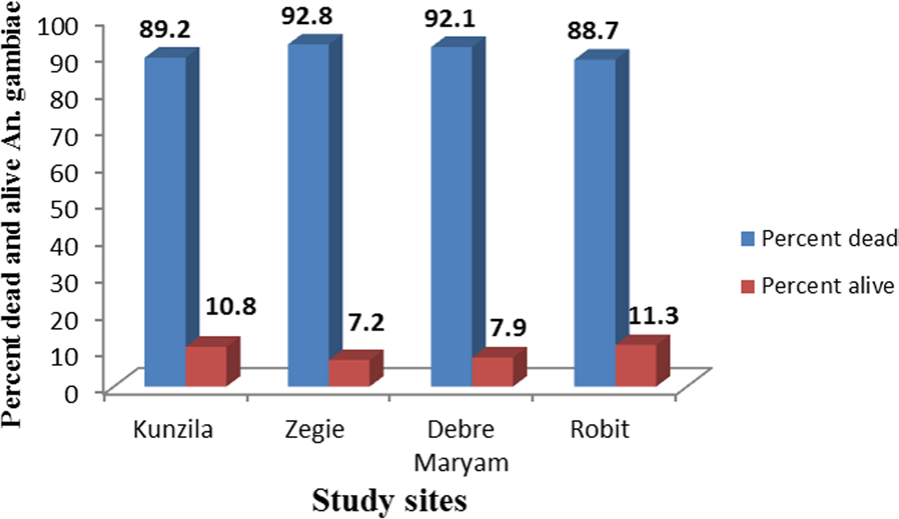
Percent of dead and resistant mosquitoes across the study areas

**Fig. 3:**
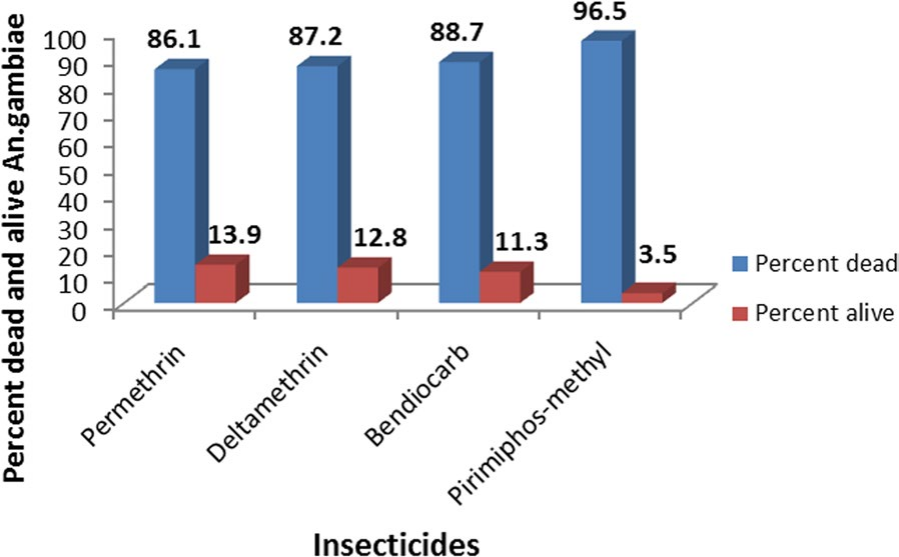
Percent of dead and resistant mosquitoes by different insecticides

Totally, 1300 *Anopheles gambiae* s.l. were tested whether they were susceptible to the four insecticides (permethrin, deltamethrin, pirimiphos-methyl and bendiocarb) or not at different study sites. Among these, 90.7% of them were susceptible to insecticides, whereas the remaining 9.3% of specimens were resistant to the insecticides (Additional file 1: Table S1). In the study sites, the most susceptible *An*. *gambiae* s.l. was found in Zegie followed by Debre Maryam, Kunzila and Robit (Fig. 2). Among the four insecticides, pirimiphos-methyl (96.5%) was the most effective to kill *An*. *gambiae* s.l. followed by bendiocarb (88.7%), deltamethrin (87.2%) and permethrin (86.1%) (Fig. 3).

According to the results of the multivariate analysis of variance, mortality significantly varied between insecticides (*F* = 26.06, *DF* = 3, *P* < 0.0001), but not between study locations (*F* = 1.56, *DF* = 3, *P* = 0.212). The interaction effect between the study site and insecticide showed the insignificant variation of *An. gambiae* s.l. mortality (*F* = 1.77, *DF* = 9, *P* = 0.10) (Table 1).

**Table 1:** Interaction effects of study sites and insecticides tested on *An*. *gambiae* s.l. across Lake Tana, northwest Ethiopia

Source of variation	Dependent variable	DF	Mean square	*F*	*p* value
Study sites	Dead	3	2.18	1.56	0.212
	Alive	3	2.89	2.06	0.118
Insecticides	Dead	3	36.52	26.06	0.000
	Alive	3	13.39	9.56	0.000
Study sites * Insecticides	Dead	9	2.47	1.77	0.100
	Alive	9	1.79	1.28	0.272

The calculated control mortality rate is less than 5% in all tests, therefore, no need of calculating the corrected mortality rate for the tested mosquitoes. The highest mortality rate (98.8%) due to pirimiphos-methyl insecticide was recorded in Zegie, whereas the least mortality rate (82.5%) due to deltamethrin insecticide was recorded in Robit. Similar trends of mortality rate were observed in Debre Maryam (97.5%) and Kunzila (85%) by bendiocarb insecticide. Deltamethrin and bendiocarb were indicated to kill more than 90% of *Anopheles* mosquitoes in Zegie, whereas pirimiphos-methyl was also indicated to kill the same percentage of *Anopheles* mosquitoes in the four study sites, such as Kunzila, Debre Maryam, Robit and Zegie. On the other hand, the death of less than 90% of *An*. *gambiae* s.l. were observed in the four sites (Kunzila, Zegie, Debre Maryam and Robit), the three sites (Kunzila, Debre Maryam and Robit), and the two sites (Kunzila and Robit), respectively, treated with permethrin, deltamethrin and bendiocarb (Table 2).

**Table 2:** Effect of different insecticides on the mortality of *An. gambiae* s.l. at different locations in Lake Tana area of Ethiopia

Insecticides	Debre Maryam	Kunzila	Robit	Zegie
Pirimiphos-methyl	96.59ab	97.62ab	95.45ab	98.75a
Bendiocarb	97.50ab	85.0bc	87.5abc	93.75abc
Deltamethrin	88.75abc	86.25abc	82.5c	91.25abc
Permethrin	85.0bc	87.5abc	88.75abc	87.5abc
Control	1.76d	1.22d	2.39d	1.88d

Means not connected by the same letter(s) are significantly different from each other according to Tukey Honestly Significance difference test at *α* = 0.05

The mean comparison of dead mosquitoes showed some signs of interaction between bendiocarb and locations. Pirimiphos-methyl had no interaction effect with the location, because it performed more or less equally across locations, i.e., 95.5–99% mortality. Bendiocarb, on the other hand, was more effective at Debre Mariam and Zegie (> 93% mortality) than at other locations (< 88% mortality), indicating some interaction with locations. The control had low mortality (< 3%) regardless of location. Insecticides in general killed more than 80% of the *An*. *gambiae* s.l. tested during the study (Table 2).

The 50% and 95% knockdown times (KDT50 and KDT95) were determined against four insecticides. In all study sites (except Debre Maryam), the fastest knockdown time (KDT50) was recorded in pirimiphos-methyl followed by bendiocarb, deltamethrin and permethrin. The fastest knockdown mosquito (KDT50 = 52.7) was recorded in Zegie by pirimiphos-methyl, whereas the slowest (KDT50 = 85.7) was recorded in Robit by permethrin. Similar trends were recorded from knockdown times (KDT95) in all study sites and insecticides used (Table 3).

**Table 3:** Knockdown times (minutes) (KDT50 and KDT95) of *An. gambiae* s.l. in different insecticides across the study areas

Study area	Insecticide	No. mosquitoes tested	KDT50 (95%, CI)	KDT95 (95%, CI)
Kunzila	Permethrin	80	80.3 (74.3–86.8)	133.5 (123.6–145.8)
	Deltamethrin	80	75.7 (70.0–82.0)	128.9 (119.4–140.8)
	Bendiocarb	80	60.5 (54.9–66.1)	113.7 (105.3–124.1)
	Pirimiphos-methyl	84	57.6 (52.2–63.0)	110.6 (102.6–120.9)
Zegie	Permethrin	80	75.3(68.6–82.6)	128.6 (117.9–142.4)
	Deltamethrin	80	72.4 (65.8–79.5)	125.7 (115.2–139.1)
	Bendiocarb	80	56.2 (49.6–62.8)	109.5 (100.2–121.3)
	Pirimiphos-methyl	80	52.7 (46.0–59.2)	105.9 (96.9–117.4)
Debre Maryam	Permethrin	80	74.6 (68.0–81.8)	128.5 (118.0–142.1)
	Deltamethrin	80	70.7 (64.1–77.6)	124.5 (114.3–137.7)
	Bendiocarb	80	57.6 (51.1–64.1)	111.5 (102.2–123)
	Pirimiphos-methyl	88	59.6 (53.5–65.9)	113 (104.3–125.3)
Robit	Permethrin	80	85.7 (79.7–92.4)	140.9 (130.6–153.9)
	Deltamethrin	80	81.0 (75.2–87.2)	136.2 (126.3–148.6)
	Bendiocarb	80	64.8 (59.570.2)	120.0 (111.4–130.8)
	Pirimiphos-methyl	88	64.6 (59.5–69.7)	119.8 (111.3–130.4)

## Discussion

The current study showed that *An*. *gambiae* s.l. susceptibility was varied in study sites and types of insecticides used which is similar to other types of studies conducted in Ethiopia [19, 32, 33]. These variations are depending on the extensive and repeated use of insecticides in the form of IRSand LLINs for vector control [34]. The use of insecticides for other purposes such as agriculture and public health could also play a role in the increase of insecticide resistance in various places [35]. For instance, *An. gambiae* s.l. mosquitoes develop resistance to pyrethroids, because these classes of insecticides are applied repeatedly for the control of household pests and vectors which is implicated in the observed selection of the high levels of kdr resistance [36]. In addition, it might be explained by the differences in their mode of action and the inherited traits of the malaria vector involved in the treatments [37].

Malaria vectors develop insecticide resistance through different mechanisms. Among these, the resistance of pyrethroid is linked with the existence of the kdr allele in mosquitoes [38]. In southwestern Ethiopia, the high frequency of the kdr allele in malaria vectors was first documented and recorded [5]. Later, the same findings from Ethiopia's north, center, and southwestern regions were recorded [25, 39].

This study showed the presence of susceptibility and possible resistance in pirimiphos-methyl-treated *An*. *gambiae* s.l. Similarly, another study showed that *An*. *arabiensis* was fully susceptible to pirimiphos-methyl in some study sites [19]. Another study conducted in Ethiopia showed that a population of *An*. *arabiensis* was fully susceptible to bendiocarb and pirimiphos-methyl [32, 33, 40]. In contrast, mosquito population resistance to pirimiphos-methyl was detected in Babile (Oromia Regional State, Ethiopia) with a mortality rate of 85% [19].

Permethrin treated *An*. *gambiae* s.l. developed resistance (< 90%) in all study sites. This report is similar to some studies carried out in Ethiopia that showed high resistance of *An*. *arabiensis* to pyrethroids (permethrin and deltamethrin) [5, 19, 32, 35, 41]. *Anopheles arabiensis* was resistant and susceptible to permethrin in Metehara and Melka Worer, respectively [23]. A similar observation of resistance in populations of *An*. *arabiensis* to permethrin had been reported from Sudan [42]. In contrast, populations of *An*. *gambiae* s.l. proved susceptible to pyrethroids at localities in the eastern parts of the country [35, 43].

In this study, resistance and possible resistance were developed in deltamethrin and bendiocarb-treated *An*. *gambiae* s.l. at all study sites. Similarly, resistance and suspected resistance to deltamethrin were reported in different parts of Ethiopia [33]. Likewise, a low level or possible bendiocarb (carbamate) resistance was detected in different study sites of Ethiopia, such as Asendabo, Bahir Dar, Chewaka, Alamata and Lare with mortality rates of 93%, 87%, 90%, 96% and 92%, respectively [19, 33]. Permethrin and deltamethrin resistance emerged in field populations of *An. arabiensis* from high-risk and low-risk areas [24]. The population of *An*. *arabiensis* from all sites were resistant to deltamethrin with mean percent mortality rates of ranged between 9% and 75% [32, 37]. On the other hand, *An*. *arabiensis* showed fully susceptible to bendiocarb [35, 37, 44]. Surprisingly, malaria vectors isolated from a region of Metema in northwest Ethiopia showed nearly complete susceptibility to deltamethrin, with an average death of 99% [19].

Depending on the type of insecticide and the location of capture, *An. gambiae* s.l. was knocked-down at different rates. This variation is due to the susceptibility status of *Anopheles* mosquitoes and the nature of insecticides used [44]. Knockdown time is affected by the concentration of insecticides, the exposure interval, and the residual time of post-application [45].

The fastest knockdown mosquito was recorded in Zegie by pirimiphos-methyl, whereas the slowest was recorded in Robit by permethrin. This is similar to studies conducted in different parts of Ethiopia and DR Congo showing different KDT50 and KDT95 in different study sites and insecticides used [32, 44]. The result of this study revealed that permethrin had the slowest knockdown times. This result is similar to some studies carried out in Ethiopia that showed KDT50 of permethrin was greater than 60 min for all *An*. *gambiae* s.l. samples from the three study sites [24, 35]. On the other hand, the KDT50 of permethrin was much less than 60 min in Ethiopia and Sudan [23, 46], which is much faster than the present study.

The KDT50 value for deltamethrin was less than 60 min for both low-risk and high-risk groups [24, 44], which is a little bit faster than the current study. However, the KDT95 of all three samples tested for deltamethrin was greater than 60 min [35], which is more or less similar to our study. Likewise, 95% of *Anopheles* were knocked after 53 min of deltamethrin treatment [44]. In addition to this, all populations from the desert and semi-desert sites showed a faster KDT50 and KDT95 to bendiocarb than to DDT and malathion [46], which is more or less similar to the current study. In bendiocarb, the KDT50 was below 60 min, whereas KDT95 was above 60 min [47]. The current study showed that pirimiphos-methyl had lower KDT50 and KDT95 than other insecticides. This report is more or less similar to the study conducted in Tanzania [48]. However, another study conducted in Ghana showed that pirimiphos-methyl had the highest knockdown times when compared to other groups of insecticides [49].

The emergence of insecticide resistance in populations of *An*. *gambiae* s.l. could threaten the current vector control operations in Ethiopia [50]. Pyrethroids were regarded to play a significant role in malaria control measures [5]. In areas where pyrethroid resistance has been detected, it affects the efficacy of control treatments, such as ITNs and IRS [11]. As a result, the observed resistance to pyrethroids and suspected resistance to bendiocarb in mosquito populations in the study area calls for ongoing resistance monitoring to delay or slow down insecticide resistance. Combining two insecticides with different modes of action has been suggested as a resistance management method that tries to kill resistant vectors [51]. In addition to this, deploying different insecticides in different regions, using insecticides in rotation manner and integrated vector management have been suggested as a resistance management method.

## Conclusions

The current study revealed that the knockdown times and effectiveness of different insecticides varied in different study sites. In Zegie, the pirimiphos-methyl was the most effective in knockdown and killing *An*. *gambiae* s.l when compared to the other insecticides and study sites. The average mortality rate of this study confirmed the presence of resistance in permethrin (87.2%) and deltamethrin (87.23%) and suspected resistance in bendiocarb (91%) and pirimiphos-methyl (97.1%). Therefore, insecticide resistance information is very essential for concerned bodies to make informed and evidence-based decisions on vector control. This study did not show the mechanisms of resistance due to a shortage of materials and chemicals as well as proper laboratory access both in our country and abroad. Hence, further studies should be conducted in these study areas to know the mechanisms of resistance specifically and the resistance levels of malaria vectors in different parts of Ethiopia broadly.

## Supplementary Information

**Additional file 1: Table S1.** Number of dead and resistant mosquitoes across the study areas by different insecticides.

## Data Availability

The entire row data are available on the request from the corresponding author.

## References

[1] Raghavendra K, Barik TK, Reddy BN, Sharma P, Dash AP (2011). Malaria vector control: from past to future. Parasitol Res.

[2] Soko W, Chimbari MJ, Mukaratirwa S (2015). Insecticide resistance in malaria-transmitting mosquitoes in Zimbabwe: a review. Infect Dis Poverty.

[3] Callaghan A. Insecticide resistance: mechanisms and detection methods. Sci Prog. (1933-). 1991:423-37.

[4] Ranson H, Jensen B, Vulule J, Wang X, Hemingway J, Collins F (2000). Identification of a point mutation in the voltage-gated sodium channel gene of Kenyan *Anopheles gambiae* associated with resistance to DDT and pyrethroids. Insect Mol Biol.

[5] Yewhalaw D, Van Bortel W, Denis L, Coosemans M, Duchateau L, Speybroeck N (2010). First evidence of high knockdown resistance frequency in *Anopheles arabiensis* (Diptera: Culicidae) from Ethiopia. Am J Trop Med Hyg.

[6] WHO. World Malaria Report 2017. Geneva, World Health Organization, 2017.

[7] WHO. Recommended insecticides for indoor residual spraying against malaria vectors. Geneva: World Health Organization; 2018.

[8] WHO. World malaria report 2021. Geneva, World Health Organization, 2021.

[9] Coetzee M, Hunt RH, Wilkerson R, Della Torre A, Coulibaly MB, Besansky NJ (2013). *Anopheles coluzzii* and *Anopheles amharicus*, new members of the *Anopheles gambiae* complex. Zootaxa.

[10] Alemayehu E, Asale A, Eba K, Getahun K, Tushune K, Bryon A (2017). Mapping insecticide resistance and characterization of resistance mechanisms in *Anopheles arabiensis* (Diptera: Culicidae) in Ethiopia. Parasit Vectors.

[11] N’Guessan R, Corbel V, Akogbéto M, Rowland M (2007). Reduced efficacy of insecticide-treated nets and indoor residual spraying for malaria control in pyrethroid resistance area, Benin. Emerg Infect Dis.

[12] FMoH. Five-year National Operational Plan for Malaria Prevention and Control in Ethiopia 2016–2020. Addis Ababa, 2016.

[13] Kyalo D, Amratia P, Mundia CW, Mbogo CM, Coetzee M, Snow RW (2017). A geo-coded inventory of anophelines in the Afrotropical Region south of the Sahara: 1898-2016 [Version 1; referees: 3 approved]. Wellcome Open Res..

[14] Irish SR, Kyalo D, Snow RW, Coetzee M. Updated list of Anopheles species (Diptera: Culicidae) by country in the Afrotropical Region and associated islands. Zootaxa. 2020;4747:zootaxa. 4747.3. 10.11646/zootaxa.4747.3.1 PMC7116328 32230095

[15] Kibret S, Yihenew A, Boelee E, Habte T, Dawit A, Beyene P (2010). The impact of a small-scale irrigation scheme on malaria transmission in Ziway area, Central Ethiopia. Trop Med Int Health.

[16] Jaleta K, Sharon HR, Seyoum E, Balkew M, Gebre-Michael T, Ignell R (2013). Agro-ecosystems impact malaria prevalence: large-scale irrigation drives vector population in western Ethiopia. Malar J.

[17] Bhatt S, Weiss D, Cameron E, Bisanzio D, Mappin B, Dalrymple U (2015). The effect of malaria control on *Plasmodium falciparum* in Africa between 2000 and 2015. Nature.

[18] Biscoe ML, Mutero CM, Kramer RA. Current policy and status of DDT use for malaria control in Ethiopia, Uganda, Kenya and South Africa: Working paper 95. IWMI; 2004.

[19] Messenger LA, Shililu J, Irish SR, Anshebo GY, Tesfaye AG, Ye-Ebiyo Y (2017). Insecticide resistance in *Anopheles arabiensis* from Ethiopia (2012–2016): a nationwide study for insecticide resistance monitoring. Malar J.

[20] Asale A, Getachew Y, Hailesilassie W, Speybroeck N, Duchateau L, Yewhalaw D (2014). Evaluation of the efficacy of DDT indoor residual spraying and long-lasting insecticidal nets against insecticide resistant populations of *Anopheles arabiensis* Patton (Diptera: Culicidae) from Ethiopia using experimental huts. Parasit Vectors.

[21] Yewhalaw D, Asale A, Tushune K, Getachew Y, Duchateau L, Speybroeck N (2012). Bio-efficacy of selected long-lasting insecticidal nets against pyrethroid resistant *Anopheles arabiensis* from South-Western Ethiopia. Parasit Vectors.

[22] Massebo F, Lindtjørn B (2013). The effect of screening doors and windows on indoor density of *Anopheles arabiensis* in south-west Ethiopia: a randomized trial. Malar J.

[23] Balkew M, Gebre-Michael T, Hailu A (2003). Insecticide susceptibility level of *Anopheles arabiensis* in two agrodevelopment localities in eastern Ethiopia. Parassitologia.

[24] Yewhalaw D, Wassie F, Steurbaut W, Spanoghe P, Van Bortel W, Denis L (2011). Multiple insecticide resistance: an impediment to insecticide-based malaria vector control program. PLoS ONE.

[25] Fettene M, Olana D, Christian R, Koekemoer L, Coetzee M (2013). Insecticide resistance in *Anopheles **arabiensis* from Ethiopia. Afr Entomol.

[26] Ranson H, N’guessan R, Lines J, Moiroux N, Nkuni Z, Corbel V (2011). Pyrethroid resistance in African anopheline mosquitoes: what are the implications for malaria control?. Trends Parasitol..

[27] WHO. Test procedures for insecticide resistance monitoring in malaria vector mosquitoes. 2016; 9241511575.

[28] Coetzee M (2020). Key to the females of Afrotropical Anopheles mosquitoes (Diptera: Culicidae). Malar J.

[29] Lemine AMM, Lemrabott MAO, Basco LK, Bogreau H, Faye O, Boukhary AOMS (2018). Pyrethroid resistance in the major malaria vector *Anopheles **arabiensis* in Nouakchott, Mauritania. Parasites Vectors.

[30] Chanyalew T, Natea G, Amenu D, Yewhalaw D, Simma EA (2022). Composition of mosquito fauna and insecticide resistance status of *Anopheles gambiae* sensu lato in Itang special district, Gambella, Southwestern Ethiopia. Malar J.

[31] Abbott WS (1925). A method of computing the effectiveness of an insecticide. J Econ Entomol.

[32] Balkew M, Ibrahim M, Koekemoer LL, Brooke BD, Engers H, Aseffa A (2010). Insecticide resistance in *Anopheles arabiensis* (Diptera: Culicidae) from villages in central, northern and south west Ethiopia and detection of kdr mutation. Parasit Vectors.

[33] PMI. President’s malaria initiative Ethiopia, Malaria Operational Plan FY 2019, 2019.

[34] WHO. World Health Organisation 2012. Global plan for insecticide resistance management in malaria vectors, 2012.

[35] Boussougou-Sambe ST, Eyisap WE, Tasse GCT, Mandeng SE, Mbakop LR, Enyong P (2018). Insecticide susceptibility status of *Anopheles gambiae* (sl) in South-West Cameroon four years after long-lasting insecticidal net mass distribution. Parasit Vectors.

[36] Weill M, Lutfalla G, Mogensen K, Chandre F, Berthomieu A, Berticat C (2003). Insecticide resistance in mosquito vectors. Nature.

[37] Abate A, Getu E, Wale M, Hadis M, Mekonen W (2020). Impact of bendiocarb 80% WP indoor residual spraying on insecticide resistance status of *Anopheles **arabiensis*. Ethiop J Sci Technol.

[38] Bloomquist JR (1996). Ion channels as targets for insecticides. Annu Rev Entomol.

[39] Balkew M, Ibrahim M, Koekemoer LL, Brooke BD, Engers H, Aseffa A (2010). Insecticide resistance in *Anopheles **arabiensis* (Diptera: Culicidae) from villages in central, northern and south west Ethiopia and detection of kdr mutation. Parasites Vectors.

[40] FDRE. Ethiopian malaria elimination plan: 2021–2025, 2020.

[41] Niang EHA, Konaté L, Diallo M, Faye O, Dia I (2016). Patterns of insecticide resistance and knock down resistance (kdr) in malaria vectors *An. **arabiensis*, *An. **coluzzii* and *An. gambiae* from sympatric areas in Senegal. Parasites Vectors.

[42] Abdalla H, Matambo T, Koekemoer L, Mnzava A, Hunt R, Coetzee M (2008). Insecticide susceptibility and vector status of natural populations of *Anopheles arabiensis* from Sudan. Trans R Soc Trop Med Hyg.

[43] Himeidan YES, Dukeen M, El Rayah EA, Adam I (2004). *Anopheles **arabiensis*: abundance and insecticide resistance in an irrigated area of eastern Sudan. EMHJ-Eastern Mediterr Health J..

[44] Matubi EM, Kaounga GI, Zanga J, Mbuku GB, Maniania JNK, Mulenda B, et al. Insecticide susceptibility of *Anopheles gambiae* sl and identification of some resistance mechanisms in Kwilu Province in the Democratic Republic of Congo. Pan Afr Med J. 2020;37(79). 10.11604/pamj.2020.37.79.18635 PMC7680223 33244342

[45] Athanassiou CG, Kavallieratos NG, Arthur FH, Nakas CT (2021). Rating knockdown of flour beetles after exposure to two insecticides as an indicator of mortality. Sci Rep.

[46] Korti M, Ageep T, Adam A, Shitta K, Hassan A, Algadam A (2021). Status of insecticide susceptibility in *Anopheles arabiensis* and detection of the knockdown resistance mutation (kdr) concerning agricultural practices from Northern Sudan state, Sudan. J Genet Eng Biotechnol.

[47] Nnko EJ, Kihamia C, Tenu F, Premji Z, Kweka EJ (2017). Insecticide use pattern and phenotypic susceptibility of *Anopheles gambiae* sensu lato to commonly used insecticides in Lower Moshi, northern Tanzania. BMC Res Notes.

[48] Mbepera S, Nkwengulila G, Peter R, Mausa EA, Mahande AM, Coetzee M (2017). The influence of age on insecticide susceptibility of *Anopheles arabiensis* during dry and rainy seasons in rice irrigation schemes of Northern Tanzania. Malar J.

[49] Akuamoah-Boateng Y, Brenyah R, Kwarteng S, Obuam P, Owusu-Frimpong I, Agyapong A (2021). Malaria transmission, vector diversity, and insecticide resistance at a peri-urban site in the forest zone of Ghana. Front Trop Dis..

[50] FMoH. National malaria elimination roadmap. National malaria prevention control and elimination. Disease prevention and control directorate. Ethiopia Addis Ababa. 2016.

[51] WHO. Global plan for insecticide resistance management. Geneva: World Health Organization; 2012.

