# Supplementary material for: Insecticide susceptibility status of Anopheles gambiae (s.l.) in and surrounding areas of Lake Tana, northwest Ethiopia
Source: Trop Med Health. 2023 Jan 13;51:3. doi: 10.1186/s41182-023-00497-w (PMC9838068; doi:10.1186/s41182-023-00497-w)
Supplement: Supplementary file 1 — Additional file 1: Table S1. Number of dead and resistant mosquitoes across the study areas by different insecticides. [file 41182_2023_497_MOESM1_ESM.doc]

**Additional file 1: Table S1.** Number of dead and resistant mosquitoes across the study areas by different insecticides

| **Study sites** | **No. of mosquitoes tested** | **No. of dead mosquitoes (%)** | **No. of alive mosquitoes (%)** |
| --- | --- | --- | --- |
| Kunzila | 324 | 289 (89.2) | 35 (10.8) |
| Zegie | 320 | 297 (92.8) | 23 (7.2) |
| Debre Maryam | 328 | 302 (92.1) | 26 (7.9) |
| Robit | 328 | 291 (88.7) | 37 (11.3) |
| **Total** | **1300** | **1179 (90.7)** | **121(9.3)** |
| **Insecticides** |  |  |  |
| Permethrin | 320 | 279 (86.1) | 41 (13.9) |
| Deltamethrin | 320 | 279 (87.2) | 41 (12.8) |
| Bendiocarb | 320 | 291(88.7) | 29 (11.3) |
| Pirimiphos-methyl | 340 | 330 (96.5) | 10 (3.5) |
